# 

**Published:** 1897-01-30

**Authors:** 


### EARLY EDITION.

**Notices of Births, Deaths, and Marriages** are inserted in this column at a charge of 2s. 6d. for an announcement not exceeding four lines.

### NOTICE TO CORRESPONDENTS.

All MS., Letters, Books for Review, and other matters intended for the Editor should be addressed The Editor, "The Hospital," The Hospital Building, 28 & 29, Southampton Street, Strand, W.C.

The Editor cannot undertake to return rejected MS., even when accompanied by stamped directed envelope.

**Notice to Advertisers and Subscribers.**

All Advertisements, Orders for Copies of The Hospital, and Business Communications should be addressed to **THE MANAGER, (Not to The Editor),** The Hospital, The Hospital Building, 28 & 29, Southampton Street, Strand, London, W.C.

The Cost of Subscription, post free, is as follows:—Per week, 2½d.; per quarter, 2s. 8d.; per half-year, 5s. 3d.; per annum, 10s. 6d. Foreign:—Per annum, 15s. 2d.; payable, in all cases, in advance.

**NOTICE.—Vol. XX. of "The Hospital" (April to September, 1896), in handsome cloth binding, is now on sale at the Office of this Journal, price 7s. 6d. Cases for binding the Numbers contained in this Volume, 1s. 6d. Index to Vol. XX., 2½d., post free.**

**The Monthly Part for February will be ready on February 1st. Price 6d., by post 8d.**

### Books Received.

The Scientific Press.

"Charity Organisation and Jesus Christ." By Charles L. Marson, M.A.

The "Times" Office.

"A Practical Scheme for Old Age Pensions." By Henry C. Burdett. Reprinted from the *Times*.

Witherby and Co.

"Lean's Royal Navy List."

Savoy Press.

"Exposures of Quackery," Vols. I. and II.

John Wright and Co. (Bristol).

"The Climate of Bournemouth." By A. Kinsey-Morgan, M.D.

T. Fisher Unwin.

"Juvenile Offenders." By William Douglas Morrison.

Scientific Press.

"Cottage Hospital Case-Book; or Register of Patients."

### The Student of Medicine.

### APPOINTMENTS.

T. S. Adair, M.B., C.M.Edin., appointed First Assistant Medical Officer to the Wadsley Asylum of the West Riding County Council. F. S. Barnett, M.R.C.S.Eng., L.F.P.S.Glasg., appointed Medical Officer for the Lewisham District of the Lewisham Union R. D. Boase, L.R.C.P.Lond., M.R.C S.Eng., reappointed Medical Officer of Health for the Penzance Port Sanitary Authority. Miss S. Coghill, L.R.C.P., L.R.C.S.Edin., appointed Registrar and Anæsthetist to the Royal Hospital for Children and Women, Waterloo Bridge Road, S E. W. R. Dix, M.B., B.S.Durh., appointed Assistant Medical Officer, Whittingham Asylum, Preston. H. Evans, M.B.Oxon., M.R.C.S.Eng., appointed *pro tem.* Medical Officer of Health to the Seaford Urban Sanitary District. E. A. Gibson, M.B., C.M.Glasg., L.M.Rotunda, appointed Outdoor Physician to the Glasgow Maternity Hospital. M. W. S. Isacke, M.R.C.S., L.R.C.P.Lond., appointed Third Assistant Medical Officer to the Wadsley Asylum of the West Riding County Council. Dr. M. B. Kay, appointed Second Assistant Medical Officer to the Wadsley Asylum of the West Riding County Council. J. St. L. Kirwan, B.A., B.Ch., B.A.O.Roy.Univ.Irel., appointed Second Assistant Medical Officer to the District Asylum, Ballinasloe. J. C. Nixon, M.B., appointed Third Assistant Medical Officer to the Menston Asylum of the West Riding County Council. J. E. Parker, M.R.C.S.Eng., L.R.C.P.I., appointed Medical Officer for the Ince District of the Wigan Union. W. J. Penfold, M B.Edin., appointed Fourth Assistant Medical Officer to the Menston Asylum of the West Riding County Council. C. G. Roberts, M.A.Camb , M.B., B.C., appointed Medical Officer of Health to the Halstead Urban District Council. Dr. Saunders, appointed Medical Officer for the Parish of Duffus. J. F. Saunders, M.R.C.S.Eng., L.S.A , appointed Medical Officer for the Catford District of the Lewisham Union. E. W. K. Scott, M.B., C.M Edin , appointed Assistant House Surgeon to the District Hospital, West Bromwich, for six months. C. A. E. Sheaf, M.R.C.P., F.R.C.S.E., appointed Government Medical Officer, Levuka, Fiji. I. Spriggs, M.B., appointed Temporary Assistant Medical Officer to the Nottingham Union. A. J. Stiles, M.D.Edin , M.R C.S.Eng., appointed Medical Officer, West Spalding District, and for the Deeping St. Nicholas District of the Spalding Union. J. T. Kitchen, M.D.Edin., appointed Resident Medical Superintendent to the Bradford Fever Hospital. J. A. Lee, M.B., C.M.Edin , appointed Medical Officer and Public Vaccinator of the Eyam District of the Bakewell Union. Mr. Black, B.A.Camb., M.R.C.S., L.R.C.P., appointed House Surgeon to the Suffolk General Hospital. T. R Bailey, M.D.Edin., appointed Medical Officer for the No. 1 District of the Parish of Bilston, of the Wolverhampton Union. L. Birch, L.R.C P.I., L.R.C.P.Edin., appointed Medical Officer and Public Vaccinator for the Ince Division of the Wigan Union.

### ROYAL NATIONAL PENSION FUND FOR NURSES.

*President*—H.R.H. THE PRINCESS OF WALES.

*Chairman*—WALTER H. BURNS, Esq. *Deputy-Chairman*—HENRY C. BURDETT, Esq.

### Statement of the Results for 1896.

| | |
|---|---|
| New Policies issued, over ... ... ... ... ... ... ... | 800 |
| Premiums received during the year, over ... ... ... ... | £55,000 |
| Pensions paid, over ... ... ... ... ... ... ... ... | £1,200 |
| Sick Pay distributed to Policy-holders, over ... ... ... ... ... | £1,100 |

Invested Funds considerably exceed £300,000.

More than £28,000 has been returned to Nurses who have been obliged to give up their Policies from one cause or another.

**The above figures speak for themselves as regards the appreciation in which the Fund is held by those for whom it was instituted.**

**Full particulars to be obtained from THE SECRETARY,**

ROYAL NATIONAL PENSION FUND FOR NURSES,

28, FINSBURY PAVEMENT, LONDON, E.C.

